# Impact of different destocking strategies on the resilience of dry rangelands

**DOI:** 10.1002/ece3.10102

**Published:** 2023-05-29

**Authors:** Toyo Vignal, Mara Baudena, Angeles Garcia Mayor, Jonathan A. Sherratt

**Affiliations:** ^1^ Department of Mathematics Heriot‐Watt University Edinburgh UK; ^2^ The Maxwell Institute of Mathematical Sciences Edinburgh UK; ^3^ Institute of Atmospheric Sciences and Climate National Research Council Turin Italy; ^4^ National Biodiversity Future Center Palermo Italy; ^5^ Copernicus Institute of Sustainable Development Utrecht University Utrecht the Netherlands

**Keywords:** adaptive management, consumer‐resource, dry rangeland, resilience

## Abstract

Half of the world's livestock live in (semi‐)arid regions, where a large proportion of people rely on animal husbandry for their survival. However, overgrazing can lead to land degradation and subsequent socio‐economic crises. Sustainable management of dry rangeland requires suitable stocking strategies and has been the subject of intense debate in the last decades. Our goal is to understand how variations in stocking strategies affect the resilience of dry rangelands. We describe rangeland dynamics through a simple mathematical model consisting of a system of coupled differential equations. In our model, livestock density is limited only by forage availability, which is itself limited by water availability. We model processes typical of dryland vegetation as a strong Allee effect, leading to bistability between a vegetated and a degraded state, even in the absence of herbivores. We study analytically the impact of varying the stocking density and the destocking adaptivity on the resilience of the system to the effects of drought. By using dynamical systems theory, we look at how different measures of resilience are affected by variations in destocking strategies. We find that the following: (1) Increasing stocking density decreases resilience, giving rise to an expected trade‐off between productivity and resilience. (2) There exists a maximal sustainable livestock density above which the system can only be degraded. This carrying capacity is common to all strategies. (3) Higher adaptivity of the destocking rate to available forage makes the system more resilient: the more adaptive a system is, the bigger the losses of vegetation it can recover from, without affecting the long‐term level of productivity. The first two results emphasize the need for suitable dry rangeland management strategies, to prevent degradation resulting from the conflict between profitability and sustainability. The third point offers a theoretical suggestion for such a strategy.

## Graphical abstract

The resilience of dry rangelands is significantly impacted by the choice of livestock management strategies, with a typical trade‐off between productivity and ecosystem resilience. Comparing two axes of variation (intensity and adaptivity) in livestock management strategies, we find that a stronger coupling between the destocking rate and the amount of forage available increases the resilience of the dry rangeland system, without decreasing its long‐term productivity.

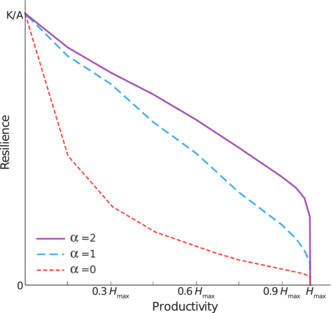

## 1 INTRODUCTION

Dry rangelands are arid, semi‐arid, and dry subhumid ecosystems that are dedicated mainly to livestock and wildlife grazing and browsing. They cover roughly a third of the Earth's land surface (ILRI et al., 2021; Safriel et al., 2006) and are home to about half of the world's livestock (Safriel et al., 2006). Water scarcity, due to a low‐precipitation‐to‐evapotranspiration ratio, limits the possibility of growing crops (Hobbs et al., 2008). Still, dry rangelands support the livelihoods of hundreds of millions of the world's poorest people through animal husbandry (de Haan, 2016; Herrero et al., 2009; Naess & Bardsen, 2013). This pivotal role in global food security is expected to increase, due to growth in both drylands' human population and their per capita consumption of meat and other animal products (de Haan, 2016; Herrero et al., 2015). Furthermore, dry rangelands provide ecosystem services such as carbon storage, soil formation, flooding control, and maintenance of biodiversity, as well as aesthetic and cultural value (FAO, 2009; Henderson et al., 2015; Sala et al., 2017; Sandhage‐Hofmann, 2016).

The first hazard dry rangelands regularly experience, independently of how they are managed, is drought. This extreme water shortage has a direct negative impact on the health and growth of dryland vegetation (Gouveia et al., 2017; NOAA et al., 2022; Vicente‐Serrano et al., 2013; Wang et al., 2015), which can be exacerbated by herbivory (Fritts et al., 2018), with subsequent negative impact on domestic herds (Catley et al., 2013; Catley et al., 2014; Naess & Bardsen, 2013). Another threat dry rangelands experience is overgrazing, which can lead to soil and vegetation degradation (Gonzalez & Ghermandi, 2021). In such contexts, drought can trigger regular collapses in animal numbers (Coppock et al., 2008; Desta & Coppock, 2002). Grazing pressure is currently intensifying due to demographic growth and other social factors affecting drylands, such as changing land use and tenure, sedentarisation of mobile pastoralists, changes in herd size and structure, and increased use of supplementary feed (Reid et al., 2014; Thornton et al., 2009). Overgrazing, in combination with drought, has been identified as a cause of ‘desertification’ (Brandt & Thornes, 1996; Geist & Lambin, 2004; Vetter, 2009; Yassoglou et al., 2017), a hardly reversible loss of productivity of dry rangelands that is also known as land degradation. There is a high confidence in the fact that desertification and climate change will cause future reductions in crop and livestock productivity (Mirzabaev et al., 2019). Accordingly, the prevention of dry rangeland degradation is a great matter of concern and research (Briske et al., 2020; Campbell et al., 2006; Jakoby et al., 2015; Sandford & Scoones, 2006; Tietjen & Jeltsch, 2007; Vetter, 2005).

A key notion in the study of dryland degradation is that of resilience of an ecosystem, that is, its capacity to maintain itself in a desirable ecological state when subjected to perturbations (Holling, 1973). In socio‐ecological systems such as dry rangelands, it is important to understand how human decisions impact the system's capacity to recover from uncontrolled perturbations. In particular, the expected increase in the frequency, duration, and intensity of droughts in many parts of the world (Cherlet et al., 2018; Vetter, 2009) calls for a better understanding of the strategies that enhance dry rangelands' resilience to drought.

**FIGURE 1:**
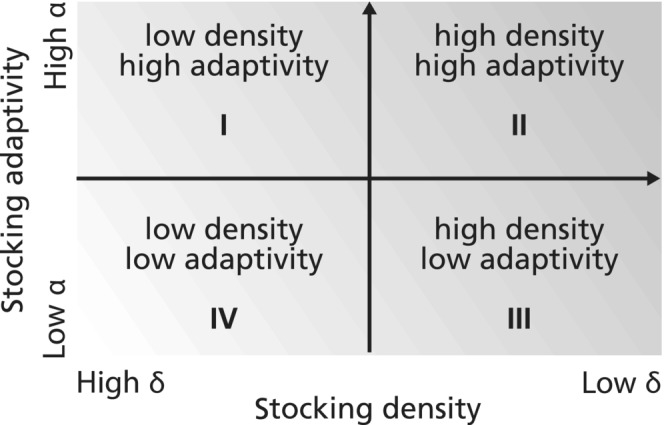
The four extreme management strategies and how they relate to our control parameters δ and α. Past research on optimal grazing strategies in dry rangelands typically opposed the poles II and IV, where II was associated with traditional pastoral systems and IV with commercial pasture management (Campbell et al., 2006). In this work, decreasing the parameter δ increases the stocking density and increasing α increases the stocking adaptivity.

In practice, different preparation and response strategies to drought are commonly implemented, with the two main strategies (Torell et al., 2010) consisting of either maintaining low stocking densities or having an adaptive (also called ‘flexible’, ‘opportunistic’, or ‘tracking’) stocking density. The former aims at keeping the number of animals low to minimize the risk of overstocking during dry events. The latter involves actively adjusting the herd size according to rainfall or available forage. Choosing whether to apply a low‐density or an adaptive stocking strategy in dry rangelands has been at the core of animated debates for decades (Campbell et al., 2006; Sandford & Scoones, 2006; Torell et al., 2010), which have not been settled yet (Sandhage‐Hofmann, 2016). These debates have given rise to a more theoretical argument on whether dry rangelands follow equilibrium or non‐equilibrium dynamics (see Briske et al., 2020; Vetter, 2005 for an overview of this issue). It is worth mentioning that the low‐density and adaptive strategies have erroneously been presented as the two opposing extremes of a spectrum, when in reality, intensity and adaptivity of stocking density are two separate axes of variation in livestock management strategies (Campbell et al., 2006), as shown in Figure 1.

This means that changes in density and adaptivity not only have distinct implications in terms of productivity and resilience of the rangeland but also that they can be combined. Another shortcoming identified by some of the actors in the debate is the lack of generality of previous studies, with many results being extrapolated from case studies (Campbell et al., 2006) or tied to particular conditions (Sandford & Scoones, 2006). Addressing these gaps, this work investigates the impact of different combinations of stocking density and adaptivity on the resilience of a generic dry rangeland using mathematical modeling.

Our model is similar to that of van de Koppel and Rietkerk (2000), which is a coupled system of ordinary differential equations (ODEs) based on pioneering work on the stability of consumer‐resource systems (Noy‐Meir, 1975; Rosenzweig & MacArthur, 1963). In contrast with these earlier models, by taking into account dryland vegetation's vulnerability to land degradation, the van de Koppel model predicts the possibility of an almost irreversible loss of vegetation and the subsequent collapse of herbivores in the system. In analogous models for temperate rangelands, such an abrupt irreversible change can be observed only if limitations other than forage availability are put on herbivore growth (Noy‐Meir, 1975). Van de Koppel et al. show that these collapses to a degraded state are less likely to happen in systems where herbivores and vegetation are coupled than in systems where the two biomasses are uncoupled (van de Koppel & Rietkerk, 2000). They conclude that adaptive management strategies may not preclude the irreversible collapse of the system if they are not rapid enough to prevent soil degradation. In a spatial simulation‐based study designed for semi‐arid rangelands, Jakoby et al. (2015) compare two stocking strategies in which herbivore growth depends fully on vegetation intake, with a fixed stocking goal in one case and a goal that depends on available forage in the other case. Their simulations suggest that a system with a high constant stocking goal is more prone to collapse than a system where the stocking goal is high but adapts to the available forage. However, even though both van de Koppel and Rietkerk (2000) and Jakoby et al. (2015) show how adaptivity of the stocking density can help avoid catastrophic shifts, neither investigates the resilience of the system to external perturbations, such as droughts.

In Fletcher and Hilbert (2007), a generic consumer‐resource model applicable to temperate rangelands is analyzed numerically to assess how different management choices can affect resilience to external perturbations. The authors show that different management strategies can yield the same long‐term level of productivity but drastically differ in terms of the resilience of the system. Like van de Koppel and Rietkerk (2000), Fletcher and Hilbert find that totally decoupling herbivore growth from vegetation can be detrimental to the vegetation and, hence, to the whole system. However, the strategies compared by Fletcher and Hilbert (2007) aggregate several types of undefined management actions with no clear mapping to real‐world processes. Moreover, animal dynamics are not modeled explicitly and do not incorporate the impact of forage availability on herbivore growth. In contrast, the present work targets a single type of managerial action that regulates herd size. In our model, herbivore growth depends exclusively on the amount of vegetation available (i.e., we do not consider supplementary feed). Therefore, the herd size is actively managed only through the removal of animals or their (re)introduction into the system. Our model focuses exclusively on destocking, the reduction of the number of animals in the system.

In practice, destocking is being increasingly used to mitigate herbivory pressure on dryland vegetation, minimize livestock die‐offs during droughts, and generate positive socio‐economic effects (Morton & Barton, 2002). A great proportion of ranchers use destocking as one of their strategies to respond to droughts (Kachergis et al., 2014; Salmoral et al., 2020), while humanitarian programs in dry, famine‐prone areas regularly facilitate emergency sales and slaughtering of livestock during droughts (Abebe et al., 2008; Aklilu & Wekesa, 2002; Morton & Barton, 2002). Existing destocking strategies differ mainly in terms of their baseline rate and their degree of adaptivity to environmental changes (Morton & Barton, 2002). As the baseline destocking rate determines the herd size, comparing these strategies links us back to the debate around low‐density versus adaptive strategies. Our aim is to investigate how both of these strategies affect the resilience of dry rangeland systems.

To our knowledge, no general model exists that explicitly defines different destocking strategies and compares their impact on dry rangelands. In this work, we formulate a broadly applicable mathematical model and explain how variations in the destocking rate impact the productivity and the resilience of the desirable steady state of a generic dry rangeland system subject to drought. Avoiding the classical polarization of the debate, we look separately at the effects of variations in stocking density and stocking adaptivity. In terms of productivity, we focus on the long‐term herd size and how it varies with changes in management parameters. In terms of resilience, we investigate how different destocking strategies affect the existence of a sustainable productive state and the threshold before a drought‐induced catastrophic collapse. We consider the possibility of, and the potential mechanisms for increasing the resilience of a dry rangeland without reducing its long‐term productivity.

## 2 METHODS

### 2.1 Model

Similarly to the model in van de Koppel and Rietkerk (2000) and the general predator–prey model in Wang et al. (2011), we represent the plants and herbivores dynamics with a comprehensive consumer‐resource system of two coupled ODEs (1) with generic functional forms under simple constraints (a1‐a6, detailed later). Conducting our analysis on such a general form means that our results hold for all the particular cases it covers. Our findings are, hence, more robust and general than the ones derived from a specific formulation.

$$\begin{array}{cc} \underset{\text{change}\;\text{in}\;\text{time}\;\text{of}\;\text{the}\;\text{vegetation}\;\text{density}}{\underbrace{\frac{dV}{dt}}}=\underset{\text{plant}\;\text{growth}}{\underbrace{f\left(V\right)}}-\underset{\text{consumption}\;\text{by}\;\text{herbivores}}{\underbrace{\mathrm{Hg}\left(V\right)}} & \left(1a\right)\\ \underset{\text{change}\;\text{in}\;\text{time}\;\text{of}\;\text{the}\;\text{herbivore}\;\text{density}}{\underbrace{\frac{\mathrm{dH}}{\mathrm{dt}}}}=\underset{\text{herd}\;\text{growth}}{\underbrace{\mathrm{\gamma Hg}\left(V\right)}}-\underset{\text{destocking}}{\underbrace{HD_{\delta ;\alpha }\left(V\right)}} & \left(1b\right) \end{array}\;$$

The non‐negative state variables V and H are the vegetation and herbivore densities, respectively. Note that H is usually called ‘stocking rate’ in rangeland management. $f\left(V\right)$ is the vegetation growth rate. $g\left(V\right)$ is the per capita consumption rate of vegetation by animals. γ is the plant‐to‐animal conversion factor, while $D_{\delta ;\alpha }\left(V\right)$ is the per capita animal loss rate that specifies how fast animals are removed from the system and how this removal varies with the quantity of forage available. $D_{\delta ;\alpha }\left(V\right)$ can represent the decay in animal biomass due to senescence, illness, and removal by human managers. In particular, $D_{\delta ;\alpha }\left(V\right)$ can represent different management strategies, depending on the value of its parameters δ>0 and α≥0. Typically, in consumer‐resource (predator–prey) models, this loss term is a constant. In our model, α is the destocking adaptivity parameter that represents the degree of coupling between the animal loss rate and the density of forage available. When α=0, the loss term $D_{\delta ;\alpha }\left(V\right)$ reduces to a constant, whose value is determined by δ. Hence, δ fixes the baseline destocking rate that sets the intensity of the per capita loss rate $D_{\delta ;\alpha }\left(V\right)$ and by doing so, fixes the long‐term stocking density. In cases where α>0, the parameter δ still fixes the baseline destocking rate and the long‐term stocking density but $D_{\delta ;\alpha }\left(V\right)$ will increase as vegetation density declines. The degree of this adaptation, consisting of removing more animals as the available forage decreases, depends on α: the greater the value of α, the greater the adaptivity of $D_{\delta ;\alpha }\left(V\right)$ to lack of forage. In practice, an increased animal mortality rate correlated to forage scarcity can be due to either natural causes (such as dehydration, illnesses, lower immunity, etc.) or a management strategy. Even though the term $D_{\delta ;\alpha }\left(V\right)$ can be interpreted as a constant or resource‐dependent loss of animals due to natural causes, we refer to it as a ‘destocking strategy’ in the rest of the text. We give a more precise form for $D_{\delta ;\alpha }\left(V\right)$ in constraint a4.

### 2.2 Constraints to the functional forms of f, g and D

**FIGURE 2:**
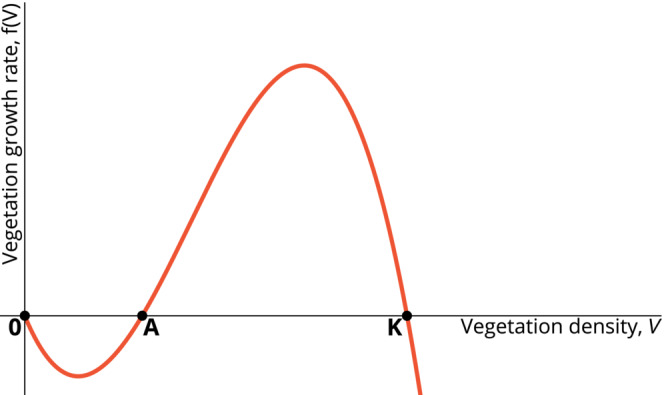
Example of vegetation growth function satisfying the constraint a1. The function given here is of the shape $f\left(V\right)=\mathrm{rV}\left(\frac{V}{A}-1\right)\left(1-\frac{V}{K}\right)$, where r is a positive constant.

Table 1 compiles the constraints we are adding to the system of equations (1). The set of constraints a1 applies to the plant growth function $f\left(V\right)$. We assume that there is a strong feedback, typical of water scarce environments, between vegetation and soil water, leading to a strong Allee effect; that is, the existence of a critical density threshold below which the population growth is negative. This is not the case for all dry rangelands, as discussed in van de Koppel and Rietkerk (2000). Ecologically, the strong Allee effect in drylands corresponds to the combined phenomena of plant–plant facilitation (Kéfi et al., 2007; Rietkerk et al., 2004) and self‐reinforcement of the bare ground (Saco et al., 2007): in (semi‐)arid regions, existing plants facilitate the growth and survival of those nearby by locally reducing evaporation and enhancing water infiltration (Davies et al., 2007; Holmgren et al., 1997; Holzapfel et al., 2006) (see Callaway (2007) for a description of the main plant–plant facilitation mechanisms), whereas bare soils are subject to increased erosion, which prevents the establishment of new plants (Saco et al., 2007). These feedback loops consolidate the bare ground state in such a way that plant growth and establishment are difficult at low vegetation densities (Courchamp, 2008). As mentioned earlier, in the case of a strong Allee effect, vegetation growth is negative below a critical density (as for example in Figure 2), called the Allee threshold. Similar to the vegetation carrying capacity K, the Allee threshold A is typically a composite parameter that depends on various environmental conditions as well as the plant species. We will see in the Results (Section 3) that the existence of this positive feedback gives rise to bistability in vegetation productivity and is, hence, associated with the possibility of a discontinuous transition between alternative steady states, even in the absence of herbivores. An abrupt transition between a productive and a degraded state corresponds to the long‐term degradation of the rangeland. Figure 2 gives an example of a function $f\left(V\right)$ that satisfies a1.

**TABLE 1:** Additional model constraints.

	Constraint	Meaning
a1	$f\left(V\right)$ is differentiable, with 3 roots: 0,A and K, such that K>A>0, $f^{\prime }\left(0\right)<0,$ $f^{\prime }\left(A\right)>0$ and $f^{\prime }\left(K\right)<0$.	Vegetation growth is continuous, A is its Allee threshold, K is its carrying capacity.
a2	$g\left(0\right)=0$	There is no foraging in the absence of vegetation.
a3	$g^{\prime }\left(V\right)>0$	Denser vegetation leads to more foraging, possibly saturating.
a4	$D_{\delta ,\alpha }\left(V\right)=X\left(\delta \right){\left(\frac{V_{\mathrm{eq}}\left(\delta \right)}{V+\varepsilon }\right)}^{\alpha }$	α sets the destocking adaptivity such that for a fixed δ, all α yield the same equilibrium.
a5	$X\left(\delta \right)>0$, $X^{\prime }\left(\delta \right)\geq 0$	δ sets the stocking density. There is no restocking: we consider only naturally rebuilding herds.
a6	ε<<1	ε is a negligible term that prevents division by 0.

Constraints a2 and a3 on the per capita consumption rate g of the herbivores ensure that the animals feed on existing vegetation and that a higher density of forage makes consumption more efficient: as the animals do not have to move as much to find food, their foraging efficiency is improved. It is noteworthy that the widely used linear, Holling II, and Holling III functional responses satisfy requirements a2 and a3 on $g\left(V\right)$.

Constraint a4 and its subconstraints a5 and a6 describe the management strategies we are studying. Our goal is to understand the influence on resilience of varying destocking strategies across the two axes of variation presented in Figure 1. The constraints on the destocking rate $D_{\delta ;\alpha }$ allow us to investigate separately the effects of the stocking density at equilibrium and of the adaptivity to changes in vegetation density. The control parameter δ sets the stocking density. By constraint a5, the destocking rate increases as δ increases. Therefore, as can be seen in the results (Section 3), the stocking density decreases as δ increases. We will also see that the vegetation density at equilibrium $V_{\mathrm{eq}}$ depends on δ. The expression $\frac{V_{\mathrm{eq}}\left(\delta \right)}{V+\varepsilon }$ compares the vegetation density at equilibrium $V_{\mathrm{eq}}$ to the current vegetation density V: the lower V is, the greater $\frac{V_{\mathrm{eq}}\left(\delta \right)}{V+\varepsilon }$ is. The control parameter α sets the intensity of destocking adaptivity by exacerbating the dependency of $D_{\delta ;\alpha }\left(V\right)$ on $\frac{V_{\mathrm{eq}}\left(\delta \right)}{V+\varepsilon }$.

Importantly, by construction, once δ is fixed, all strategies $D_{\delta ;\alpha }\left(V\right)$ give rise to the same long‐term herd size $H_{\mathrm{eq}}$, no matter the value of α. We show in the Results (Section 3) that this means that long‐term production is determined by δ exclusively, and is equivalent for all values of α. Constraint a4 imposes that $D_{\delta ;\alpha }\left(V\right)$ is positive, and either a constant (α=0) or increasing as vegetation density declines (α>0).

Note that we assume that the per capita animal loss rate $D_{\delta ;\alpha }\left(V\right)$ is purely a management decision and that we neglect the loss of animal biomass due to metabolic expenses, to simplify our analysis. Importantly, in the case of a constant metabolic expense rate, as is generally found in the literature, adding such a term, that is, rewriting $D_{\delta ;\alpha }\left(V\right)$ as $m+D_{\delta ;\alpha }\left(V\right),$ with m>0 does not affect our results, provided that

$$\lim_{V\to \infty }\;\mathrm{\gamma g}\left(V\right)>m,$$

that is, provided that the animal growth rate when vegetation is unlimited is greater than the metabolic expense rate.

In addition to constraints a1‐a6, there are several implicit constraints that are already enclosed in the system of coupled equations (1):

- Because animal growth depends on vegetation only, there is no other source of calories for the livestock, that is, no supplementary feed.
- Herd growth is limited by food availability only, not by space limitation or other density‐dependent factors. This assumption is motivated by the fact that dry rangelands are typically extensive exploitation systems, where herd growth is limited by forage availability, which is itself limited by water availability.
- The dynamics described are spatially homogeneous and continuous in time, with constant environmental parameters A and K. The stable steady states represent the possible long‐term configurations of the system. Perturbations of the state variables can be applied to represent the effects of below and above average conditions, as well as exceptional events that are external to the model. This is discussed in more detail in the ‘resilience’ paragraph below. The model can apply to ranchers and pastoralists but ignores herd mobility by averaging the dynamics over space. Because it also assumes uniform management, that is, a single given destocking strategy over the whole domain, the model is suitable for one management unit, such as one ranch or one uniformly managed pastoral area. The model can, hence, apply to a wide range of unit sizes, from tens to thousands of hectares.

### 2.3 Analysis

Our goal is to compare the resilience of a productive system under different degrees of stocking density and adaptivity, that is, for different values of the stocking density parameter δ and of the adaptivity parameter α. For this, we first need to understand under which conditions a system is productive and how to measure its resilience to the effects of drought, which we model as a perturbation as described in the following. Then, we can compare two systems with identical parameters except for the control parameter of interest, namely δ or α. Similarly to classical work on consumer‐resource systems (Noy‐Meir, 1975; Rosenzweig & MacArthur, 1963), we use linear stability and graphical analysis of the phase plane (defined below) to both understand the range of possible behaviors of the system and define resilience metrics.

### 2.4 Phase plane analysis

The phase plane is the space of all possible states $\left(V;H\right)$ of the system and their evolution, for a given parametrisation. It features the solution trajectories and, in particular, the steady states of the system (1). Each point $\left(V;H\right)$ can represent an initial condition of (1) that will evolve following a unique solution trajectory governed by the equations. We use the phase plane to summarize the stability results and explain:

- in which cases vegetation and herbivory are theoretically incompatible and why this is so.
- how different destocking strategies affect the resilience of a productive state.

To draw the phase plane, we need to consider the nullclines, that is, the sets of points for which one of the two populations does not change. These curves, solutions to the equations $\frac{\mathrm{dV}}{\mathrm{dt}}=0$ and $\frac{\mathrm{dH}}{\mathrm{dt}}=0,$ (which yields the two vegetation and the two herbivore nullclines, respectively), partition the phase plane into areas of different behaviors. The steady states of the system lie at the intersections of a vegetation and herbivore nullcline.

### 2.5 Sustainable productive state

A sustainable productive state of the system is defined as a steady state $\left(V_{\mathrm{eq}};H_{\mathrm{eq}}\right)$ that is stable and strictly positive in its two components. We refer to its *H*‐component, $H_{\mathrm{eq}}$, as the ‘long‐term stocking density’ or the ‘long‐term stocking goal’ of the system. Although positive stable limit cycles are possible, we exclude them from our analysis, as managers are unlikely to try to achieve a fluctuating herd size.

### 2.6 Resilience of a sustainable productive state

**FIGURE 3:**
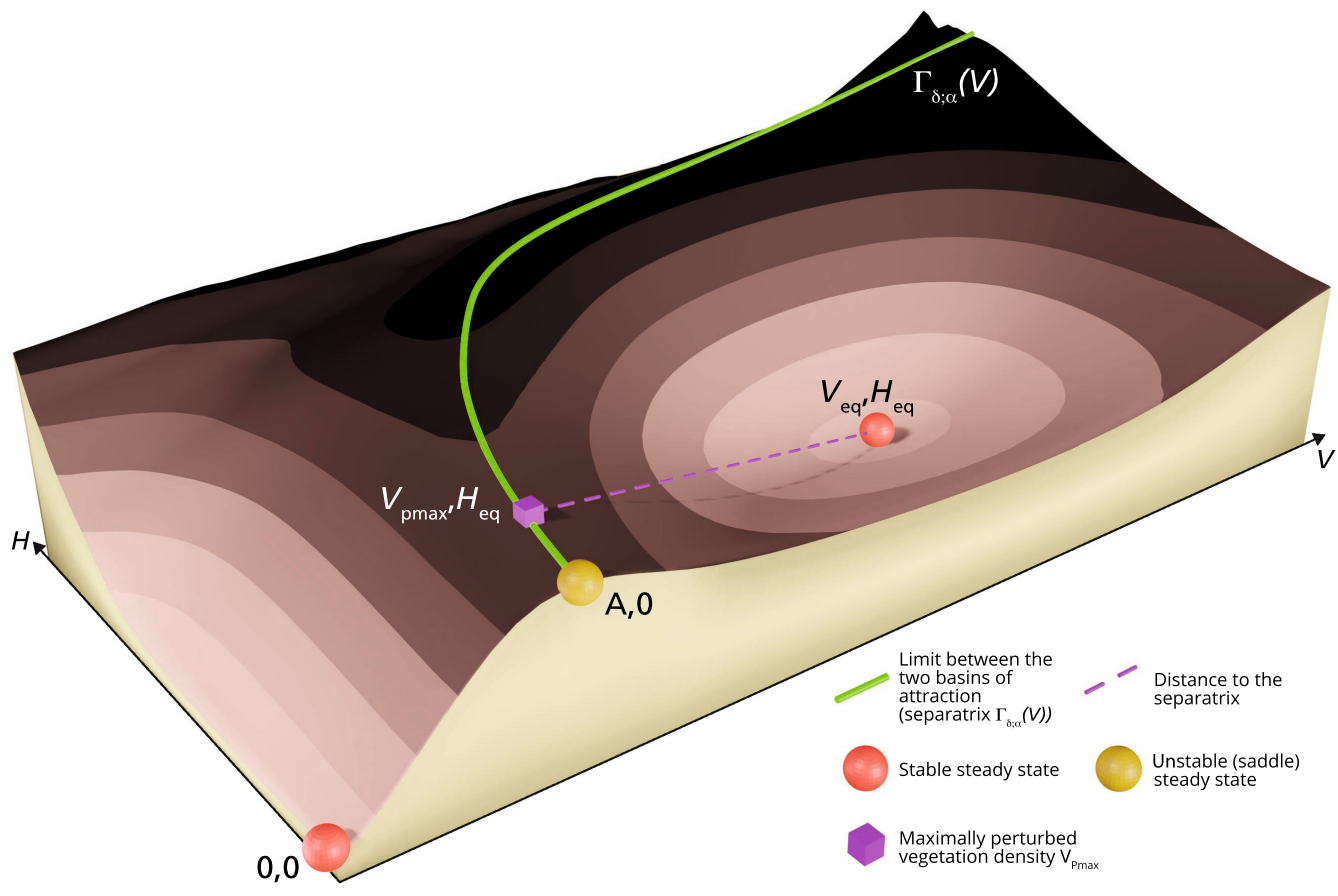
Qualitative 3D stability landscape of our two dimensional system. The separatrix (green continuous line) marks the limit between the basin of attraction of the sustainable productive steady state $\left(V_{\mathrm{eq}};H_{\mathrm{eq}}\right)$ and the basin of attraction of the degraded state (0;0). The position of the separatrix depends on the destocking strategy.

When the system is at a sustainable productive state, it can either recover fully or shift to a less desirable equilibrium after a state variable perturbation. As mentioned previously, there exists a threshold in the phase plane, beyond which the system cannot recover to the stable productive state. This threshold, also known as the ‘separatrix’, is the boundary between the degraded and the productive states' basins of attraction (see Figure 3). In other words, the long‐term impact of a perturbation on the system depends on whether or not it has sent the state variables outside the basin of attraction of the stable productive state (van de Koppel & Rietkerk, 2000; van Voorn et al., 2007). The position of the separatrix depends on the values of the parameters and, hence, on the management strategy adopted. In extreme cases, a change in parameters can cause a stable steady state to lose its stability. This critical event corresponds to a bifurcation of the system. Importantly, the boundaries of a stable state's basin of attraction can be used to design metrics of that state's resilience to perturbations (Dakos & Kéfi, 2022; Krakovská et al., 2021), as explained in the next paragraph.

### 2.7 Resilience metrics

The concept of resilience, although central in dynamical systems, is not unequivocally characterized: there exist a variety of definitions and measures of a system's resilience (Dakos & Kéfi, 2022; Krakovská et al., 2021; Rotz & Fraser, 2015). We define here two different measures of the resilience of the desirable steady state for given management parameters δ and α. Each of the measures captures a different aspect of resilience.

- The distance to bifurcation is a measure of the resilience of the system to changes in parameter values. It is defined as the minimum amount of change needed along a given parameter to cause the disappearance of the desirable (sustainably productive) steady state (Dakos & Kéfi, 2022). It, therefore, measures how much management strategies are allowed to change, even gradually, before the system would collapse. We study it for both parameters. First, for changes in the parameter δ, we write ${\text{dist}}_{\mathrm{bif}\alpha }\left(\delta \right)$, defined, for a fixed α value, as

$${\text{dist}}_{\mathrm{bif}\;\alpha }\left(\delta \right)=|\delta -\delta_{\mathrm{bif}}|,$$

where δ is the current δ value and $\delta_{\mathrm{bif}}$ is the value of δ at which the closest bifurcation occurs. When considering changes in the parameter α, we write ${\text{dist}}_{\mathrm{bif}}\delta \left(\alpha \right)$. It is defined for a fixed δ, as

$${\text{dist}}_{\mathrm{bif}\;\delta }\left(\alpha \right)=|\alpha -\alpha_{\mathrm{bif}}|,$$

where α is the current α value and $\alpha_{\mathrm{bif}}$ is the value of α at which the closest bifurcation occurs.

- The maximal loss ratio $\frac{V_{\mathrm{eq}}}{V_{\text{pmax}}}\left(\delta ;\alpha \right)$ is a measure of the resilience of the desirable steady state to external perturbations. It reflects how many times greater the vegetation density at equilibrium is, compared with the vegetation density at which the tipping point to the degraded state occurs. $V_{\text{pmax}}\left(\delta ;\alpha \right)$ is the minimal value of V still in the basin of attraction of the productive steady state, when no change in H is applied (see below for a description of the perturbation and Figure 3 for an illustration of $V_{\text{pmax}}$). In practice, $V_{\text{pmax}}\left(\delta ;\alpha \right)$ is the lowest the vegetation can get, due to an external perturbation (such as a drought), without the system collapsing to the degraded state.

### 2.8 Modeling drought as a perturbation

We model the effects of drought as a sudden reduction of vegetation, with no change in H. This assumes a rapid effect of drought on vegetation (Noy‐Meir, 1975; Zhao et al., 2020). The perturbed state, therefore, has the form $\left(V_{\text{pert}};H_{\mathrm{eq}}\right)$, where the perturbed vegetation state $V_{\text{pert}}$ is such that $V_{\text{pert}}<V_{\mathrm{eq}}$. The perturbation is impulsive (pulse perturbation) in the sense that we set the initial conditions of the system (1) to a perturbed state, then the system is subject to its usual dynamics. The perturbation is isolated in the sense that we always allow the system to recover or degrade fully after the perturbation is applied.

## 3 RESULTS

In this section, we first describe the range of behaviors the model system (2.1) can exhibit and specify the conditions of existence and stability of a productive state. Then, we compare the productivity and the resilience to drought of a sustainable productive state under different destocking strategies. A model example with realistic specification and parametrisation, described in the appendix, is used to generate the figures. The purpose of this model example is purely illustrative, as all results are derived analytically, independently of any model specification or parametrisation. Their validity depend on the satisfaction of constraints a1‐a6 only.

### 3.1 General behavior of the system (phase plane analysis)

**FIGURE 4:**
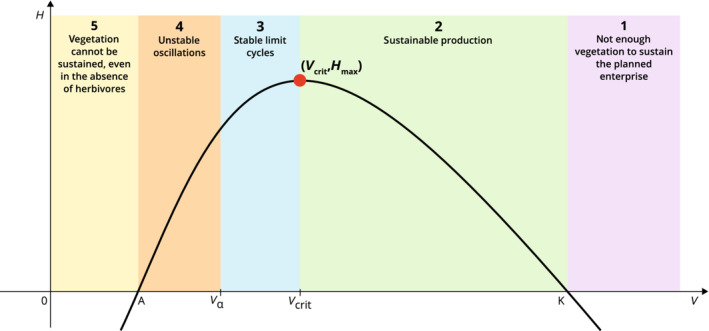
Graphical summary of the general stability analysis, as a phase plane. Note that the vertical management nullcline $\mathrm{\gamma g}\left(V\right)-D_{\delta ;\alpha }\left(V\right)=0$ is not shown, as its position varies with δ. Decreasing (resp. increasing) δ moves the management nullcline to the left (resp.right). The phase plane is partitioned into five areas (labeled 1–5) where the vertical management nullcline can lie, and for each of which the system displays qualitatively different behavior.

**FIGURE 5:**
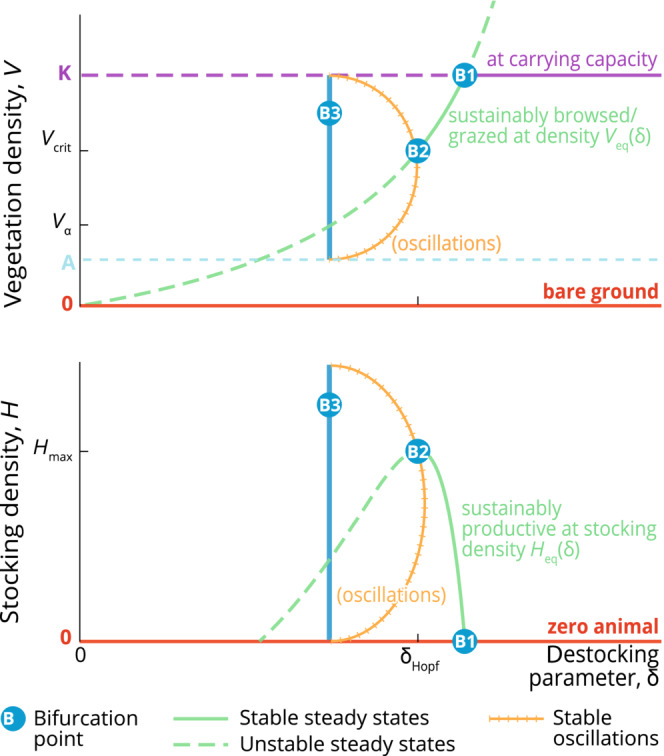
Bifurcation diagrams for vegetation density (top panel) and animal density (bottom panel): as the destocking parameter δ (horizontal axis) varies, the system undergoes transitions in its stability properties.

Our model is a general consumer‐resource (predator–prey) system with a strong Allee effect on the resource. Equations of this type have been studied analytically in Wang et al. (2011) for the case where the consumer's mortality rate (i.e., our destocking rate) is constant, that is, α=0. Our analysis showed that the behavior of the system, illustrated in Figures 4 and 5, is qualitatively the same for all other α values.

Figure 4 represents the different possible phase planes and how the system's properties vary depending on the position of the nullclines. First, we have the two trivial nullclines H≡0 (coinciding with the horizontal V‐axis) and V≡0 (coinciding with the vertical H‐axis), respectively, indicating that there is no growth of animals or plants in the absence of their conspecifics. Then, we have the non‐trivial, hump‐shaped, vegetation nullcline $ℋ\left(V\right)=\frac{f\left(V\right)}{g\left(V\right)}$. The nullcline $ℋ\left(V\right)$ splits the phase plane into the area above it, where vegetation density decreases, and the area below it, where vegetation density increases. Finally, we have the non‐trivial herbivore nullcline $D_{\delta ;\alpha }\left(V\right)=\mathrm{\gamma g}\left(V\right)$. This equation is independent of H because our model assumes that growth and destocking both depend linearly on herbivore density. Moreover, the shape of the functions $D_{\delta ;\alpha }(V)$ and γ(V) mean that the equation has a unique solution. The nullcline is therefore a vertical line at the value of V given by this unique solution, which depends on parameter values, and most significantly on the baseline destocking parameter δ. As we vary δ through our analysis, the management nullcline's position is not shown in the figure. Decreasing δ shifts this nullcline to the left, while increasing δ shifts it to the right. The intersection between $ℋ\left(V\right)$ and the management nullcline gives the potentially productive steady state $\left(V_{\mathrm{eq}};H_{\mathrm{eq}}\right)$. Depending on where the intersection happens, the system has different stability properties, which are represented by the different colored zones (1–5).

We start by describing properties of the phase plane that are common to all five phase plane configurations. Regardless of the destocking strategy in place, the system always admits three herbivore‐free steady states. These correspond to the steady states of the plant‐only model, with a strong Allee effect growth. First, the two trivial nullclines V≡0 and H≡0 intersect at $\left(0;0\right)$, the fully degraded steady state with bare ground and no animals. Then, the hump‐shaped vegetation nullcline $ℋ\left(V\right)=\frac{f\left(V\right)}{g\left(V\right)}$ intersects H≡0 in $\left(A;0\right)$ and $\left(K;0\right)$ no matter the shape and values of $D_{\delta ;\alpha }\left(V\right)$. None of these three equilibria is desirable from our rangeland management perspective, as they all feature a long‐term herbivore density equal to zero. The only possible desirable steady state will occur at the intersection of $ℋ\left(V\right)$ and the management nullcline $D_{\delta ;\alpha }\left(V\right)=\mathrm{\gamma g}\left(V\right)$. From the shapes of the non‐trivial nullclines, we see that, for a given parametrisation, there can be at most one sustainable productive steady state, $\left(V_{\mathrm{eq}};H_{\mathrm{eq}}\right)$. This stable node or sink is a potential management goal, with $H_{\mathrm{eq}}$ the long‐term stocking target of the rangeland manager. Note that with our definition of $D_{\delta ;\alpha }$, for any given δ, the management nullclines coincide for all values α. On the other hand, varying the value of δ shifts the position of the nullcline: toward the right as we increase δ and toward the left as δ is decreased. Hence, the values of $V_{\mathrm{eq}}$ and $H_{\mathrm{eq}}$ as well as the stability properties of the four steady states $\left(0;0\right),$ $\left(A;0\right)$, $\left(K;0\right)$, and $\left(V_{\mathrm{eq}};H_{\mathrm{eq}}\right)$ depend on the value of δ, while they remain unchanged by variations in α. There are five broad categories of behavior:

**region (1)** $V_{\mathrm{eq}}>K$. Because it implies that $H_{\mathrm{eq}}$ is negative and that the equilibrium $\left(V_{\mathrm{eq}};H_{\mathrm{eq}}\right)$ is unstable, this configuration is not ecologically relevant. Depending on the initial conditions: either the system settles into to the vegetation only equilibrium $\left(K;0\right)$, or it degrades itself to $\left(0;0\right)$ if the initial density of herbivores was too high and/or the initial plant density too low.
**region (2)** $V_{\text{crit}}<V_{\mathrm{eq}}<K$, where $V_{\text{crit}}$ is the maximal argument of $ℋ\left(V\right)$, that is, $ℋ\left(V_{\text{crit}}\right)$ is the maximum value of the function $ℋ\left(V\right)$. Therefore, $ℋ\left(V_{\text{crit}}\right)$ is by definition the animal carrying capacity $H_{\max}$, that is, the maximal herbivore density such that the productive steady state is stable. Importantly, $H_{\max}$ is a fixed value that does not depend on the management function $D_{\delta ;\alpha }\left(V\right)$ but only on the biological functions $f\left(V\right)$ and $g\left(V\right).$ Because the equilibrium $\left(V_{\mathrm{eq}};H_{\mathrm{eq}}\right)$ is strictly positive and stable, this is a desirable configuration. The system will stabilize at the productive state $\left(V_{\mathrm{eq}};H_{\mathrm{eq}}\right)$, provided that the initial conditions lie within its basin of attraction. As mentioned earlier, the animal density at equilibrium $H_{\mathrm{eq}}$ can be thought of as a long‐term ‘stocking goal’ of the rangeland manager. The higher the stocking goal, the lower the vegetation density at equilibrium $V_{\mathrm{eq}}$, until the threshold value $\left(V_{\text{crit}};H_{\max}\right)$ is met. This point is a Hopf bifurcation, meaning a point of critical transition in the behavior of the system, where the productive equilibrium loses its stability and (stable or unstable) limit cycles appear. Such bifurcation at the top of the vegetation nullcline hump is not specific of a system with a strong Allee effect. It has been shown for other consumer‐resource systems (e.g., Hilker & Schmitz, 2008).
**region (3)** Distinguishing region 3 from region 4 is relevant only if the Hopf bifurcation abovementioned is supercritical, that is, gives rise to stable limit cycles. When the Hopf bifurcation is instead subcritical, region 3 does not exist and the dynamics are directly the ones described for region 4. Whether or not the bifurcation is supercritical or subcritical and, therefore, whether or not the oscillations are stable or unstable, depends on the specification of g and *f*. The details of the conditions for the Hopf cycle to be supercritical and, hence, for the existence of a region 3 are given in Wang et al. (2011). When this is the case, the management nullcline $D_{\delta ;\alpha }\left(V\right)=\mathrm{\gamma g}\left(V\right),$ intersects $ℋ\left(V\right)$ such that $V_{\alpha }<V_{\mathrm{eq}}<V_{\text{crit}}$. Immediately after the Hopf bifurcation, for each value of α, there is a range $\left[V_{\alpha };V_{\text{crit}}\right]$ of values of $V_{\mathrm{eq}}$ such that the system admits a stable limit cycle. Even though the productive steady state $\left(V_{\mathrm{eq}};H_{\mathrm{eq}}\right)$ is now unstable, the system is still productive, with vegetation and animal densities oscillating in time, provided that the initial values lie in the basin of attraction of this attracting cycle. Such stable oscillations can be interpreted as a sign of overexploitation of the system (van Voorn et al., 2007). They are sometimes referred to as ‘boom‐and‐bust’ cycles and have been observed episodically in ecosystems (Desta & Coppock, 2002); even leading to a successful prediction of the next crash in animal numbers (Coppock et al., 2008). As the management nullcline is shifted to the left, that is, toward $V_{\alpha },$ the amplitude of the cycle gets bigger, until H reaches 0. We call “$V_{\alpha }$” the critical value for $V_{\mathrm{eq}}$ that corresponds to the stable cycle's destruction. Below $V_{\alpha }$, stable limit cycles do not exist anymore. The destruction of this stable attractor corresponds to a global bifurcation, which marks the deterministic tipping from a productive system to the degraded state, that can be driven by changes in management.
**regions (4) and (5)** These cases are undesirable, as the only stable steady state—and hence, the only attractor—is $\left(0;0\right)$. When the management nullcline is in these regions, the whole phase plane is the basin of attraction of the degraded state. This occurs when animals are not removed fast enough from the system or, put differently, the vegetation is too fragile for this type of exploitation. Region (4) corresponds to the system collapsing due to overexploitation, whereas in region (5) the vegetation is unable to sustain itself even in the absence of herbivores.

Figure 5 is a bifurcation diagram where we summarize how the steady states' values and stability properties depend on δ. In particular, it shows the shifts and transitions in vegetation and animal densities as δ varies. We observe stable (full lines) and unstable (dashed lines) steady states, as well as stable limit cycles (crossed lines). Bifurcations occur at B1, B2, and B3. Bifurcation B1 corresponds to the transition between regions 1 and 2 in Figure 4; bifurcation B2 corresponds to the transition between regions 2 and 3; bifurcation B3 corresponds to the transition between regions 3 and 4.

### 3.2 Comparing the productivity of different destocking strategies

When considering livestock productivity, two main categories can be considered, namely systems of primary production, that involve slaughtering the animals for meat and other animal products, and systems of secondary production, in which the animals are kept alive while by‐products such as dairy and wool are harvested. We consider the impact of varying the stocking density and adaptivity on these two production systems.

For a dairy production system with a given δ and any α, we assume that the dairy production at equilibrium during the arbitrary time interval [0;t] is proportional to the number of animals so that production will be equal to

$$\int_{0}^{t}\kappa H_{\mathrm{eq}}\left(\delta \right)\mathrm{ds}=\mathrm{t\kappa}H_{\mathrm{eq}}\left(\delta \right),$$

where κ is a constant reflecting the per capita dairy production rate.

In the case of a meat production system with a given δ and any α, the meat production at equilibrium during the arbitrary time interval $\left[0;t\right]$ is proportional to the sum of all the animals removed from the system during that interval, that is,

$$\int_{0}^{t}D_{\delta ;\alpha }\left(V_{\mathrm{eq}}\left(\delta \right)\right)H_{\mathrm{eq}}\left(\delta \right)\mathrm{ds}=\int_{0}^{t}X\left(\delta \right){\left(\frac{V_{\mathrm{eq}}\left(\delta \right)}{V_{\mathrm{eq}}\left(\delta \right)+\varepsilon }\right)}^{\alpha }H_{\mathrm{eq}}\left(\delta \right)\mathrm{ds}\approx \mathrm{tX}\left(\delta \right)H_{\mathrm{eq}}\left(\delta \right),$$

assuming that all animals are slaughtered before their natural death.

We see that in both cases, the production output at equilibrium has no dependence on α. Furthermore, in both cases, the production increases as the long‐term stocking density $H_{\mathrm{eq}}$ increases (i.e., as δ decreases).

### 3.3 Comparing the resilience to drought of different destocking strategies

The resilience to degradation induced by management choices, that is, changes in the values of the parameters δ and α, is measured by the metrics ${\text{dist}}_{{\mathrm{bif}}_{\alpha }}\left(\delta \right)$ and ${\text{dist}}_{{\mathrm{bif}}_{\delta }}\left(\alpha \right)$ (defined in Section 2), respectively. A first pathway to rangeland degradation occurs when the management choice of a long‐term stocking density (dictated by the value of parameter δ) leads to the deterministic collapse of the system. This corresponds to overexploitation‐driven degradation: if the management nullcline is shifted to the left of $V_{\text{crit}}$ (in case of a subcritical Hopf bifurcation) or $V_{\alpha }$ (in case of a supercritical Hopf bifurcation), even in a gradual manner, then $\left(0;0\right)$ becomes the only stable steady state. Because the vegetation density $V_{\text{crit}}$ at which the Hopf bifurcation happens is independent of α and δ, and because we know that decreasing δ shifts the equilibrium vegetation density $V_{\mathrm{eq}}$ toward $V_{\text{crit}}$, whereas α does not affect $V_{\mathrm{eq}}$, we can conclude for the distance to bifurcation (for fixed δ and α, respectively) that

$$\begin{array}{c} {\text{dist}}_{{\mathrm{bif}}_{\delta }}\left(\alpha_{c}\right)={\text{dist}}_{{\mathrm{bif}}_{\delta }}\left(\alpha_{a}\right)\;\text{for}\;\text{any}\;\alpha_{c},\alpha_{a},\delta .\\ {\text{dist}}_{{\mathrm{bif}}_{\alpha }}\left(\delta_{h}\right)<{\text{dist}}_{{\mathrm{bif}}_{\alpha }}\left(\delta_{l}\right)\;\text{when}\;\delta_{l}>\delta_{h},\text{for}\;\text{any}\;\alpha . \end{array}$$

In other words, increasing the stocking goal $H_{\mathrm{eq}}$ brings the system closer to a deterministic shift toward an oscillating or degraded system, whereas changing the adaptivity of the system has no effect.

The second theoretical pathway to rangeland degradation, which is always present, even in case of sensible management, is a consequence of perturbations to the state variables. When the parameters and initial conditions are such that the system is in a sustainable productive state $\left(V_{\mathrm{eq}};H_{\mathrm{eq}}\right)$, there is still a risk that external perturbations drive it to the stable degraded state $\left(0;0\right)$. This risk will vary, depending on the values of the management parameters δ and α. The resilience of a system to an external perturbation consisting of a sudden loss of vegetation (as described in Section 2), as a function of the management parameters δ and α, is measured through the resilience metric $\frac{V_{\mathrm{eq}}}{V_{\text{pmax}}}\left(\delta ;\alpha \right)$ (also described in Section 2). Deriving our results for this resilience metric takes the form of a mathematical proof. The interested reader can find explanations of unfamiliar concepts in any good linear algebra or introduction to dynamical systems textbook. Readers who are uninterested in the mathematical proof can directly go from here to the summary of the results.

To derive the resilience metric $\frac{V_{\mathrm{eq}}}{V_{\text{pmax}}}\left(\delta ;\alpha \right)$, we need to study the threshold that separates the degraded and productive states' basins of attraction in the phase plane. In our case, this curve, the ‘separatrix’, is the stable manifold of the saddle point $\left(A;0\right)$, that is, $\left(A;0\right)$'s only incoming trajectory. The separatrix can be seen as a different function of V, for each pair of parameter values α and δ, so we denote it $\Gamma_{\delta ;\alpha }\left(V\right)$. To understand where the separatrix lies in the phase plane, we first study its behavior near $\left(A;0\right),$ through linearisation. The Jacobian at $\left(A;0\right)$ is

$$J\left(A,0\right)\left(\begin{array}{cc} f^{\prime }\left(A\right) & -g\left(A\right)\\ 0 & \mathrm{\gamma g}\left(A\right)-D_{\delta ;\alpha }\left(A\right) \end{array}\right)$$

The eigenvalues are the diagonal elements $f^{\prime }\left(A\right)$ and $\mathrm{\gamma g}\left(A\right)-D_{\delta ;\alpha }\left(A\right).$ On the one hand, we have $f^{\prime }\left(A\right)>0$ (constraint a1). On the other hand, because we are considering a sustainable productive system, we know that our management nullcline is in region 2 and, hence, to the right of the point $\left(A;0\right)$. Therefore, we have $\mathrm{\gamma g}\left(A\right)-D_{\delta ;\alpha }\left(A\right)<0$, so $\left(A;0\right)$ is a saddle. A stable eigenvector is given by

(2) $$\left(1;\frac{f^{\prime }\left(A\right)+D_{\delta ;\alpha }\left(A\right)-\mathrm{\gamma g}\left(A\right)}{g\left(A\right)}\right).$$

Recalling the signs of the eigenvalues and constraints a2 and a3, we see that the second component of the eigenvector is positive. It admits a greater value, so the eigenvector is steeper, for greater values of $D_{\delta ;\alpha }\left(A\right)$. This eigenvector approximates the separatrix's behavior near $\left(A;0\right)$. Then, we can ‘go upstream’ along the trajectory $\Gamma_{\delta ;\alpha }\left(V\right)$, further away from the saddle point, by considering the equation

(3) $$\frac{\mathrm{dH}}{\mathrm{dV}}=\frac{\mathrm{\gamma Hg}\left(V\right)-D_{\delta ;\alpha }\left(V\right)H}{f\left(V\right)-\mathrm{Hg}\left(V\right)},$$

derived from the original system 1.

Now, we can derive results for our resilience metrics when α is fixed and for $\delta_{h}$, $\delta_{l}$, such that $\delta_{h}<\delta_{l}$ and such that their respective equilibria $\left(V_{{\mathrm{eq}}_{h}};H_{{\mathrm{eq}}_{h}}\right)$ and $\left(V_{{\mathrm{eq}}_{l}};H_{{\mathrm{eq}}_{l}}\right)$ are sustainably productive. We know from the previous subsection that $H_{{\mathrm{eq}}_{h}}>H_{{\mathrm{eq}}_{l}}$ and $V_{{\mathrm{eq}}_{h}}<V_{{\mathrm{eq}}_{l}}$. The stable eigenvectors are given by

$$\left(1;\frac{f^{\prime }\left(A\right)+\delta_{i}{\left(\frac{V_{\mathrm{eqi}}}{A}\right)}^{\alpha }-\mathrm{\gamma g}\left(A\right)}{g\left(A\right)}\right),i=h,l,$$

where we have ignored the infinitesimally small parameter ε since A>0 (by assumption). We have $\delta_{l}V_{{\mathrm{eq}}_{l}}^{\alpha }>\delta_{h}V_{{\mathrm{eq}}_{h}}^{\alpha }$, so the stable eigenvector is steeper in the system with $\delta_{l}$ than in the system with $\delta_{h}$. Then, Equation (3) becomes

(4) $$\frac{\mathrm{dH}}{\mathrm{dV}}=\frac{\mathrm{\gamma Hg}\left(V\right)-\delta_{i}{\left(\frac{V_{\mathrm{eqi}}}{A}\right)}^{\alpha }H}{f\left(V\right)-\mathrm{Hg}\left(V\right)},i=h,l.$$

As long as we are on the left‐hand side of the management nullclines, the numerator is negative. Because we are considering trajectories coming into $\left(A;0\right)$, and because we know that below the hump‐shaped vegetation nullcline the trajectories move away from $\left(A;0\right)$, all separatrices $\Gamma_{\delta ;\alpha }$ necessarily lie above the vegetation nullcline. Hence, the denominator is also negative. We can then conclude that the ratio (4) is positive for i=h,l, and is greater for the system with the larger δ value. We have, therefore, shown

$$\Gamma_{\delta_{l};\alpha }\left(V\right)>\Gamma_{\delta_{h};\alpha }\left(V\right)$$

for all V on the left‐hand side of the management nullcline, that is, the separatrix of the system with $\delta_{l}$ is always on the “outside” of the separatrix of the system with $\delta_{h}$ on the left‐hand side of the management nullcline. We can, hence, conclude, for any fixed α and any $\delta_{h}$, $\delta_{l}$ producing stable productive equilibria and such that $\delta_{h}<\delta_{l}$, that

$$V_{\text{pmax}}\left(\delta_{l};\alpha \right)<V_{\text{pmax}}\left(\delta_{h};\alpha \right),$$

that is, the threshold for vegetation density before collapse is lower for a greater δ, therefore

$$\frac{V_{{\mathrm{eq}}_{l}}}{V_{\text{pmax}}\left(\delta_{l};\alpha \right)}>\frac{V_{{\mathrm{eq}}_{h}}}{V_{\text{pmax}}\left(\delta_{h};\alpha \right)},$$

that is, a productive system with greater δ can recover from greater percentages of vegetation loss.

Now that we have compared the resilience of a sustainable productive system for a fixed α and different values of δ, we consider the case where δ is arbitrarily fixed such that it yields a sustainable productive system, and we have $\alpha_{c}$ and $\alpha_{a}$ such that $\alpha_{a}>\alpha_{c}$. Noticeably, following our definition, for a fixed δ, all values of α yield the same management nullcline and hence the same long‐term stocking goal $H_{\mathrm{eq}}.$ Following the same reasoning as previously, we find that

$$V_{\text{pmax}}\left(\delta ;\alpha_{a}\right)<V_{\text{pmax}}\left(\delta ;\alpha_{c}\right),$$

that is, the threshold for vegetation density before collapse is lower for a greater α, therefore

$$\frac{V_{\mathrm{eq}}}{V_{\text{pmax}}\left(\delta ;\alpha_{a}\right)}>\frac{V_{\mathrm{eq}}}{V_{\text{pmax}}\left(\delta ;\alpha_{c}\right)},$$

that is, a productive system with greater α can recover from greater percentages of vegetation loss.

### 3.4 Summary of the results for the maximal loss ratio

We have shown that, for any fixed α and any $\delta_{h}$, $\delta_{l}$ producing stable productive equilibria and such that $\delta_{h}<\delta_{l}$,

$$V_{\text{pmax}}\left(\delta_{l};\alpha \right)<V_{\text{pmax}}\left(\delta_{h};\alpha \right),$$

that is, the threshold for vegetation density before collapse is lower for a greater δ, therefore

$$\frac{V_{{\mathrm{eq}}_{l}}}{V_{\text{pmax}}\left(\delta_{l};\alpha \right)}>\frac{V_{{\mathrm{eq}}_{h}}}{V_{\text{pmax}}\left(\delta_{h};\alpha \right)},$$

that is, a productive system with greater δ can recover from greater percentages of vegetation loss.

We have also shown that, for any fixed δ producing a stable productive equilibrium and any $\alpha_{c}$, $\alpha_{a}$ such that $\alpha_{c}<\alpha_{a}$,

$$V_{\text{pmax}}\left(\delta ;\alpha_{a}\right)<V_{\text{pmax}}\left(\delta ;\alpha_{c}\right),$$

that is, the threshold for vegetation density before collapse is lower for a greater α, therefore

$$\frac{V_{\mathrm{eq}}}{V_{\text{pmax}}\left(\delta ;\alpha_{a}\right)}>\frac{V_{\mathrm{eq}}}{V_{\text{pmax}}\left(\delta ;\alpha_{c}\right)},$$

that is, a productive system with greater α can recover from greater percentages of vegetation loss.

**FIGURE 6:**
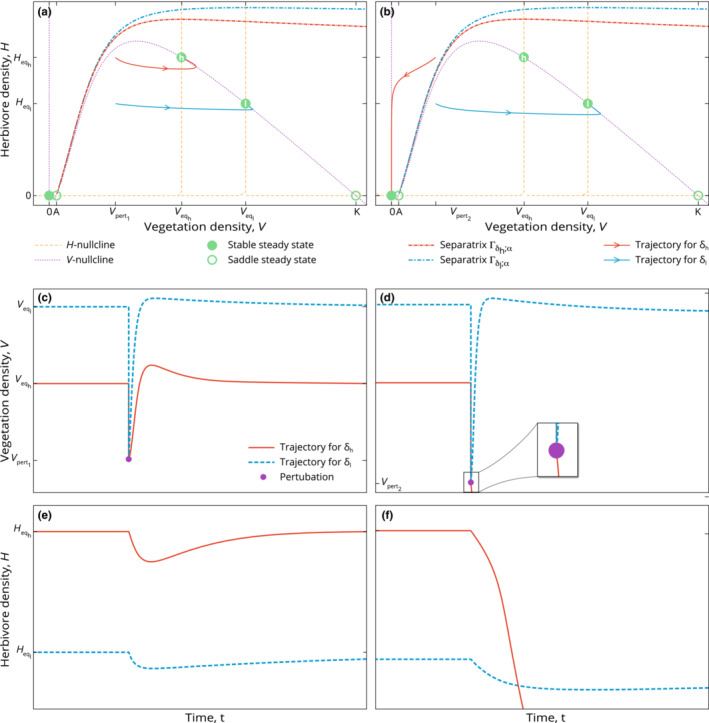
Comparing the resilience of a sustainable productive system to the effects of drought for different baseline destocking rates $\delta_{l}$ and $\delta_{h}$, where $\delta_{h}<\delta_{l}$ and with fixed adaptivity α=0. Left panels (a, c, e) show the response to a weak perturbation while right panels (b, d, f) show the response to a stronger perturbation. Top panels (a,b) show the phase‐space while the middle and bottom (c, e, d, f) show the trajectories of the biomass variables over time.

**FIGURE 7:**
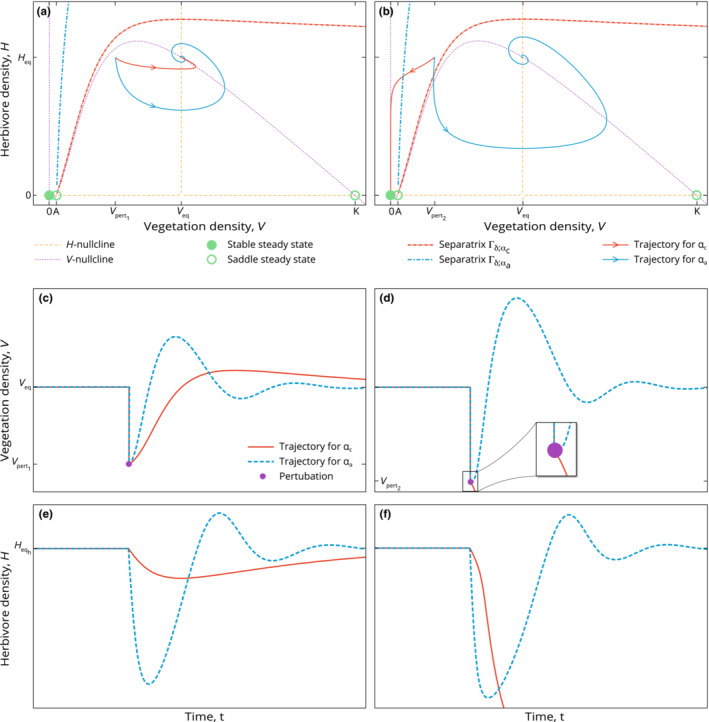
Comparing the resilience of a sustainable productive system to the effects of drought for different degrees of adaptivity $\alpha_{c}=0$ and $\alpha_{a}=1$, where the baseline destocking rate $\delta =\delta_{h}$ and hence the long‐term stocking density $H_{\mathrm{eq}}$ is fixed. Left panels (a, c, e) show the response to a weak perturbation while right panels (b, d, f) show the response to a stronger perturbation. Top panels (a,b) show the phase‐space while the middle and bottom (c, e, d, f) show the trajectories of the biomass variables over time.

In conclusion, on the one hand, a greater δ value (and therefore, a lower long‐term stocking goal *H*_*eq*_) provides greater resilience to the sustainable productive state, when considering perturbations in the negative‐*V* direction. We can conclude that for any given degree of adaptivity α, systems with lower long‐term stocking goal $H_{\mathrm{eq}}$ have greater resilience in virtue of the expected trade‐off between resilience and productivity in grazing systems. On the other hand, we have also demonstrated that greater adaptivity of the destocking rate provides greater resilience of the productive system to vegetation losses. These two results mean that increasing δ or α will systematically decrease the risk of collapse to a degraded state, as illustrated in Figures 6 and 7.

### 3.5 Illustration of the results

We generated Figures 6 and 7 using the realistic model specification and parametrisation given in the appendix. This example is derived from the well studied Klausmeier model (Klausmeier, 1999) for dryland vegetation, which consists of a system of two partial differential equations, one for plants and one for water, as explained in the appendix.

As noted earlier, the choice of parameters is for illustration purposes only and has no bearing on the general results shown above, which are valid for all parameters. However, we note that the value of $H_{\max}$ yielded by our model, $H_{\max}\approx 0.25\;\text{Tropical Livestock Units}\;\left(\mathrm{TLU}\right)\;{\mathrm{ha}}^{-1}$, is very close to the value of 0.3 TLU found in Meshesha et al. (2019), for a region where the mean annual rainfall is 200–400 mm year^−1^. The values of A and K of real‐life ecosystems are hard to assess, however the lowest mean vegetation density recorded for that same region is 0.0105 kg dry mass m^−2^ and the highest is 0.231 kg dry mass m^−2^ (Meshesha et al., 2019), which appears broadly consistent with our output values for A and K, (our parametrisation yields A≈0.0307 kg dry mass m^−2^, K≈1.303 kg dry mass m^−2^).

We compare numerically the behavior and resilience of this specific system, using the pplane8 phase plane plotter package of Matlab, (Harvey, 2022), with the Dormand–Prince (ode45) solver, for:

- two different δ values (at fixed α=0): we pick $\delta_{h}$ and $\delta_{l}$ such that they, respectively, yield the stocking targets $H_{{\mathrm{eq}}_{h}}=0.9H_{\max}$ and $H_{{\mathrm{eq}}_{l}}=0.6H_{\max}$ (Figure 6).
- two different α values (at fixed δ = $\delta_{h}$, as defined just above): we pick $\alpha_{c}$ = 0 and $\alpha_{a}$ = 1, yielding, respectively, what we call “constant” and “adaptive” strategies (Figure 7).

We apply to all four scenarios two perturbations with different magnitudes, namely the weaker perturbation pert_1_ and the stronger perturbation pert_2_. In Figure 7, pert_1_ is defined as the loss of 50% of the vegetation at equilibrium, whereas pert_2_ is defined as the loss of 66% of the vegetation at equilibrium. In Figure 6, the equilibria for $\delta_{l}$ and $\delta_{h}$ do not coincide, and pert_1_ (respectively, pert_2_) is defined as the loss of 50% (respectively, 66%) of $V_{{\mathrm{eq}}_{h}}$, the vegetation density at equilibrium associated to $\delta_{h}$. Therefore, in the higher δ case, $\delta_{l}$, these perturbations are even more severe, which strengthens our results according to which higher δ values provide more resilience to sudden losses of vegetation.

Each subfigure is the superposition of the phase planes of the two strategies we are comparing. In all cases, the vegetation nullcline $ℋ\left(V\right)$ (dotted pink) has the same position, since it is affected by neither the value of δ nor *α*. When δ is fixed, the management nullclines (dotted yellow lines) of the two parametrisations also coincide (Figure 7). The full green dots represent the stable steady states. Again, they coincide if and only if the parametrisations feature the same value for δ. The dashed lines are the separatrices, that is, the limits of the basins of attraction of the sustainable productive states. The full arrowed lines represent the trajectories of the system after a perturbation. In Figure 6, we are comparing the grazing systems' trajectories for $\delta_{h}$ and $\delta_{l}$ (α=0 is fixed). We note that the greater δ value $\delta_{l}$ yields a lower stocking target $H_{{\mathrm{eq}}_{l}}$ and that the basin of attraction for $\delta_{l}$ englobes the one for $\delta_{h}$. In subfigures (a) and (b), the equilibrium points $\left(V_{{\mathrm{eq}}_{l}};H_{{\mathrm{eq}}_{l}}\right)$ and $\left(V_{{\mathrm{eq}}_{h}};H_{{\mathrm{eq}}_{h}}\right)$ are marked with a green circle containing the letters ‘*l*’ and ‘*h*’, respectively. In subfigures (a), (c), and (e), we see that after a perturbation consisting of halving the $V_{{\mathrm{eq}}_{h}}$ vegetation density (pert_1_), both systems recover to their respective equilibria. In both cases the system was still inside the basin of attraction of its productive state after the perturbation, as can be seen in subfigure (a). In subfigures (b), (d), and (f), after a perturbation consisting of dividing by three the $V_{{\mathrm{eq}}_{h}}$ vegetation density (pert_2_), only the system with greater destocking parameter $\delta_{l}$ and lower stocking target $H_{{\mathrm{eq}}_{l}}$ recovers. The system with $\delta_{h}$ ends up degraded as the perturbation kicked the system out of the basin of attraction of $\left(V_{{\mathrm{eq}}_{h}};H_{{\mathrm{eq}}_{h}}\right)$. We see in (b) that the shape of the two separatrices in the direction of perturbation does not differ widely and we can infer from the plots (d) and (f) that the system with $\delta_{l}$ owes its recovery to its lower stocking target $H_{{\mathrm{eq}}_{l}}$. This illustrates the trade‐off between resilience and productivity in grazing systems.

In Figure 7, we are comparing the constant ($\alpha_{c}=0$) and adaptive ($\alpha_{a}=1$) destocking strategies, for a fixed $\delta =\delta_{h}$. In both cases the system has the same stable productive equilibrium $\left(V_{\mathrm{eq}};H_{\mathrm{eq}}\right)$ and, therefore, the same stocking target and productivity. In subfigures (a), (c), and (e), after a perturbation consisting of halving the $V_{\mathrm{eq}}$ vegetation density, both systems recover to $\left(V_{\mathrm{eq}};H_{\mathrm{eq}}\right)$. In both cases the system was still inside the basin of attraction of its productive state after the perturbation. In the case of the adaptive strategy $\alpha_{a}$, we can tell from the shape of the trajectory in (e) that the herd size has fluctuated more, due to a faster destocking rate after the perturbation. This could appear as a downside of this strategy. However, in subfigures (b), (d), and (f), after a perturbation consisting of dividing by three the $V_{\mathrm{eq}}$ vegetation density, only the adaptive system recovers. The system with constant destocking rate $\alpha_{c}$ ends up degraded as the perturbation moved the system out of the basin of attraction of $\left(V_{\mathrm{eq}};H_{\mathrm{eq}}\right)$. This time, the increased adaptivity of the $\alpha_{a}$ system saved it from degradation by destocking fast enough after the perturbation. Comparing panels (a) and (b) of Figure 7 with their counterparts in Figure 6, note how increasing α drastically affects the position of the separatrix, without changing the long term productivity of the system.

**FIGURE 8:**
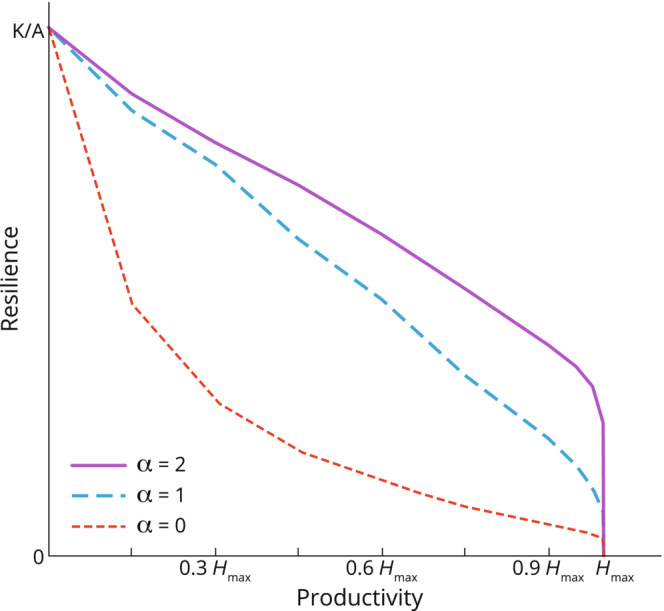
Relation between the resilience and the productivity of a sustainable productive system, for different values of the management parameters δ and α. On the horizontal axis, the long‐term stocking density $H_{\mathrm{eq}}\left(\delta \right)$ is a proxy for the rangeland's productivity. On the vertical axis, the resilience is measured through the maximal loss ratio, $\frac{V_{\mathrm{eq}}}{V_{\text{pmax}}}\left(\delta ;\alpha \right)$.

Figure 8 summarizes our main results. The curves illustrate the relationship between the resilience and the productivity of a sustainable productive dry rangeland for three different values of α. We plot the resilience metrics $V_{\text{pmax}}\left(\delta_{l};\alpha \right)$ against the herd sizes $H_{\mathrm{eq}}\left(\delta \right)$, which is a proxy for productivity. The variation in productivity shown on the horizontal axis corresponds to a variation in δ: greater baseline destocking rates δ lead to lower long‐term stocking densities $H_{\mathrm{eq}}$ and conversely. The negative slope of all three curves depicts how the resilience decreases as the productivity increases, for a given degree of adaptivity degree α. We have shown in all generality that when α is increased, the resilience increases without any loss of long‐term productivity, which is illustrated in the figure through the improvement in resilience achieved when increasing the adaptivity of the destocking rate: for all long‐term productivity values within $\left(0;H_{\max}\right)$, the curve is greater for greater values of α. For all degrees of destocking adaptivity, the resilience of the system is maximal when there are no herbivores consuming the vegetation and is minimal when the productivity is at herbivore carrying capacity $H_{\max}$, that is, at the system's Hopf bifurcation. We saw in the general results (Section 3) that the resilience decreased as $H_{\mathrm{eq}}$ increased, which can be observed for all three curves. We also saw that the resilience increased as adaptivity increased, which is illustrated through the improvement in resilience as α is increased.

## 4 DISCUSSION

A main result from this work was that dry rangelands with coupled plant‐herbivore dynamics can benefit greatly, in terms of their resilience and without loss of long‐term productivity, from adaptivity of the stocking density to the lack of forage. We found that both a low stocking density and a high stocking adaptivity, achieved through modulation of the destocking rate, provided crucial resilience in dry rangeland systems subject to drought.

Our modelling approach was very general and relied on simple and realistic constraints. The analysis is, therefore, valid for a variety of more specific models that fall under its wide umbrella. The results are robust and independent from parametrisation and from specific and possibly arbitrary choices of functional forms to represent specific mechanisms.

For any given strategy, our model results showed that a higher long‐term stocking target implies a lower resilience to sudden vegetation losses. This trade‐off between productivity and resilience is well known and observed in various exploitation models (Fletcher & Hilbert, 2007; Noy‐Meir, 1975) and is intuitively justified by the additional stress put on vegetation by a higher number of animals. Mathematically, in our work, this translated into the basin of attraction of a productive state contracting as the long‐term stocking target increases. Consistent with earlier work on general herbivore‐vegetation systems (Noy‐Meir, 1975), we found that there exists a maximal sustainable livestock density, that is, an animal carrying capacity, $H_{\max}$. The existence of an animal carrying capacity and of a trade‐off between productivity and resilience is consistent with recommendations for low stocking densities, as stocking well below carrying capacity lessens the risks of degradation following a drought (McLeod, 1997; Vetter, 2005). This trade‐off also highlights the potentially deleterious effects on resilience of strategies aiming to increase productivity, such as feed supplementation (Müller et al., 2015), or improvement in veterinary care, which decreases animal mortality in times of drought and, therefore, reduces the degree of coupling between vegetation and animals. A better understanding of these effects is essential to prevent counterproductive policies or practices. Maximizing resilience instead of short‐term productivity is also in accordance with recommendations from current frameworks of food system resilience (Rotz & Fraser, 2015).

Policy makers for sustainable rangeland management have often favored fixed stocking densities rather than traditional adaptive pastoralism, which has been perceived as a source of degradation (Lv et al., 2019). Our destocking strategies with α>0 fell into the category of adaptive stocking strategies, as they regulated the number of animals in reaction to perturbations in the vegetation density. We found two results supporting the use of adaptive strategies in dry rangelands. First, we found that the animal carrying capacity $H_{\max}$ did not vary with different degrees of adaptivity of the destocking rate: no matter how sensitive the destocking rate was to vegetation changes, all strategies allowed the same maximal number of animals in the system. Second and importantly, a higher dependence of the destocking rate on the lack of forage systematically increased the resilience of the productive state, while maintaining the long‐term production goal. This means that adaptivity in the destocking rate enabled circumventing the usual trade‐off between productivity and resilience. This result is in line with previous, less general, studies highlighting the importance of adaptivity in dry rangelands (Freier et al., 2014; Jakoby et al., 2015). As suggested by van de Koppel and Rietkerk (2000) and unlike Jakoby et al. (2015), we did not find that adaptivity eliminated the risk of a catastrophic collapse due to overstocking. We found that the higher the adaptivity, the closer resilience was to the no‐herbivore system's resilience, which itself is susceptible—by construction of our model—to irreversible drought‐driven collapses.

Our results also contributed to the theoretical debate on dry rangeland modeling, which opposes supporters of the equilibrium and non‐equilibrium theories. The latter argue that even though a carrying capacity exists, the frequency and intensity of droughts routinely force the system away from it and, therefore, make it irrelevant. In this framework, dry rangelands are, therefore, considered not to be vulnerable to overgrazing‐driven degradation, precisely because the animal density will drop after a drought (Ellis & Swif, 1988), due to decreased vegetation but also increased thirst and illness related mortality (Catley et al., 2014). This argument agrees thoroughly with our result: increased adaptivity, that is, a sharp decrease in the number of animals following a drought, will make dry rangelands more resilient to drought.

Our general results apply to dry rangelands, but they can also be applied to other consumer‐resource (or even predator–prey) systems, if the consumer's (or predator's) loss rate depends on the amount of resources available. The realistic assumption that resource shortage is correlated to consumer's weakening and lower resistance to diseases, which lead to extra mortality (Catley et al., 2014), is not accounted for in other models where, instead, the consumer loss rate is constant. We are aware of only one other predator–prey model where the predator mortality rate depends on prey density (Minter et al., 2011). In that work, which concerns protozoa, a prey density dependent death rate is derived experimentally for the predator and leads to quantitatively significant differences in the resulting dynamics. The authors show the importance of taking into account such variability in the predator's (consumer's) death rate to improve population models. In our case, we saw that resource‐dependent consumer loss rate can be a management tool that enhances the system's resilience.

Our study contained several limitations and shortcomings that could be addressed in future research. First, our model was a mean‐field representation of a system that is spatially extended in the real world. Not modeling explicitly the spatial dimension prevented our system from displaying spatial organization characteristics that are crucial in many dryland ecosystems. For instance, spatially explicit mathematical models of dryland vegetation elucidate how dryland vegetation, characterized by local plant–plant facilitation mechanisms and longer‐range competition for water, self‐organizes into patterns (Kéfi et al., 2007; Klausmeier, 1999). Importantly, according to theoretical work by Rietkerk et al. (2021), this patterning allows vegetation to evade the abrupt degradation predicted by our mean‐field model, by undergoing a more gradual change instead. However, when modeling herbivory, it has been shown that grazing can attenuate the buffering provided by spatial patterning against sudden degradation (Siero et al., 2019). Therefore, it is unclear how incorporating the spatial organization of dryland vegetation would affect our results.

Accounting for the spatial dimension would also allow for the representation of animal mobility. Nevertheless, our model already enabled us to infer that mobility was beneficial in terms of resilience to drought: resilience increased if animals disappeared from the system when vegetation density was low. This disappearance could correspond to animal displacement. The animal density could then be restored once vegetation had recovered, which would correspond to the removed animals coming back or new animals being restocked.

We could adapt our current model to better represent pastoral mobility and the practices of transhumance or rotational grazing by explicitly modeling space in a discrete manner, by defining several different pastures. Such an approach would take into account the dynamics of the displaced animals and of the alternative pasture(s), whose coupling could affect the dynamics of the original pasture, for example by acting as key resources (Illius & O'Connor, 1999; von Wehrden et al., 2012). A study on rotational grazing (Chen & Shi, 2018) finds that both production yields and stockpiled forage increase for many rotational configurations. Interestingly, there is an increasingly positive perception of pastoral mobility among dryland researchers (Adriansen, 2005) and recent studies aim at incorporating traditional indigenous knowledge into policy making (Selemani, 2020). This is consistent with recent modeling research showing that grazing, when managed in a spatially non‐uniform manner, can improve resilience to droughts (Zelnik et al., 2021).

Future research might also incorporate different modalities of environmental perturbations. Our model used a constant rainfall rate, which allowed us to focus on fluctuations in herd size that were driven by animal–plant feedback rather than by rainfall stochasticity. However, this assumption is unrealistic, as real‐life (semi‐)arid regions usually experience high variability in their rainfall rates, and these play quite some role in their vegetation dynamics (Baudena et al., 2007; Verwijmeren et al., 2021). Still, even though we did not directly model intra‐ and interannual variation in rainfall and hence in vegetation growth, the mathematical analysis of our model proved that if random pulse perturbations are applied, then lower stocking densities and higher adaptivity of the destocking rate systematically decrease the probability of degradation. Further work could study sequences of droughts that allow only partial recovery between the perturbations, or press perturbations, where the duration of the perturbation is prolonged in time, rather than modeled as an instantaneous event.

Finally, our study lacked an economic dimension since it did not take into account destocking costs or market considerations. Even though adaptive destocking did not imply any long‐term reduction in productivity, we can expect that in reality it would be associated with increased logistic costs. A more realistic bioeconomic model would need to consider market fluctuations as well as the transient production linked to extra destocking, when computing the productivity of a system. For example, a case study in the Sahel reveals how livestock owners' decision to sell—and hence their ecological impact—depends on their type of access to the market, as well as complex institutional and cultural factors Turner and Williams (2002). This study emphasizes the need to understand and incorporate local livestock market specificity when designing rangeland management policies in drought‐prone areas.

Water‐scarce and drought‐prone rangelands are vulnerable ecosystems that were traditionally managed sustainably thanks to livestock mobility (Freier et al., 2014). Now, in times of increased sedentarisation and intensification of stocking densities (Reid et al., 2014), understanding the positive impact of adaptivity on the resilience of vulnerable systems can help in the design of sustainable management strategies and policies. In practice, our study supports initiatives that facilitate adaptive destocking actions and/or livestock mobility. Initiatives to facilitate adaptive destocking actions already exist and include facilitating slaughtering at the onset of drought, easing access to the market, and transforming and giving value to destocked meat, for example, implementing logistics to dry and/or can destocked meat and distribute it as a supplement in times of drought (Abebe et al., 2008; Morton & Barton, 2002).

## AUTHOR CONTRIBUTIONS

**Toyo Vignal:** Conceptualization (lead); formal analysis (lead); methodology (lead); writing – original draft (lead). **Mara Baudena:** Conceptualization (supporting); methodology (supporting); supervision (equal); writing – review and editing (equal). **Angeles Garcia Mayor:** Conceptualization (supporting); methodology (supporting); supervision (equal); writing – review and editing (equal). **Jonathan A. Sherratt:** Conceptualization (supporting); formal analysis (supporting); methodology (supporting); supervision (equal); writing – review and editing (equal).

## FUNDING INFORMATION

UK Research and Innovation—Engineering and Physical Sciences Research Council (UKRI EPSRC) grant EP/S023291/1; Heriot‐Watt University; University of Edinburgh.

## Data Availability

Data sharing is not applicable to this article as no datasets were generated or analyzed during the current study.

## APPENDIX A Model example

### A.1

To illustrate our general model, we provide a specific example that satisfies the general form and all constraints on (1):

(5a)
$$\frac{\mathrm{dV}}{\mathrm{dt}}=\overset{f\left(V\right)}{\overbrace{\frac{{\mathrm{jrV}}^{2}R}{{\mathrm{rV}}^{2}+e}-\mathrm{mV}}}-\overset{g\left(V\right)}{\overbrace{\frac{\mathrm{\xi V}}{V+\beta }}}H$$

(5b)
$$\frac{\mathrm{dH}}{\mathrm{dt}}=\gamma \frac{\mathrm{\xi V}}{V+\beta }H-\underset{D_{\delta ;\alpha }\left(V\right)}{\underbrace{\delta {\left(\frac{V_{\mathrm{eq}}}{V}\right)}^{\alpha }}}H,$$

where V is the aboveground biomass density, j the yield of plant biomass per unit water consumed, r the rate of water uptake by plants, m the plant mortality rate, R the constant rainfall rate and e the rate of evaporation and loss to the deeper ground, ξ the maximal consumption rate per herbivore and β the half persistence parameter.

It explicitly captures how plants locally increase water availability compared with bare ground and features implicit competition for water. The non‐zero roots of $f\left(V\right)$ are

$$A≔\frac{\mathrm{jR}-\sqrt{j^{2}R^{2}-4m^{2}\frac{e}{r}}}{2m}\;\text{and}\;K≔\frac{\mathrm{jR}+\sqrt{j^{2}R^{2}-4m^{2}\frac{e}{r}}}{2m},$$

which illustrate the dependence of the vegetation carrying capacity K and Allee threshold A on environmental and plant‐specific parameters. In particular, here A and K are, respectively, a decreasing and an increasing function of the average rainfall rate R.

#### A.1 Derivation of the model

The spatially uniform version of Klausmeier model equation (Klausmeier, 1999) is

Assuming that root‐water infiltration dynamics occur on a much faster time‐scale than vegetation growth/decay and grazing dynamics, we can apply a quasi steady state approximation. Hence we suppose that available water is always at equilibrium so

$$\frac{\mathrm{dW}}{\mathrm{dt}}=R-{\mathrm{rWV}}^{2}-\mathrm{eW}=0,$$

which yields

$$W_{\mathrm{eq}}=\frac{R}{{\mathrm{rV}}^{2}+e}.$$

Substituting this in the vegetation equation yields

$$\frac{\mathrm{dV}}{\mathrm{dt}}=\frac{{\mathrm{jrV}}^{2}R}{{\mathrm{rV}}^{2}+e}-\mathrm{mV}.$$

By adding coupled herbivores dynamics where we define $g\left(V\right)$ as the widely used Holling II functional response, we have the resulting example system (5).

**TABLE A1:** Parameters of the model example (Equations 5a and 5b).

Parameter	Value	Unit	Meaning	Source
j	0.003	$\frac{\mathrm{kg}\;\mathrm{dry}\;\text{mass}}{\mathrm{kg}\;H_{2}O}$	The yield of plant biomass per unit water consumed	Klausmeier (1999)
r	100	$\frac{\mathrm{kg}\;H_{2}O\;m^{-2}\;{\text{year}}^{-1}}{{\left(\mathrm{kg}\;\mathrm{dry}\;\text{mass}\;m^{-2}\right)}^{2}}$	Rate of water uptake by plants	Klausmeier (1999)
m	0.9	year^−1^	Plant mortality rate	Siero et al. (2019)
e	4	year^−1^	Evaporation rate	Klausmeier (1999)
R	400	$\frac{\mathrm{kg}\;H_{2}O}{m^{2}\;\text{year}}$ $\left(=\mathrm{mm}\;{\text{year}}^{-1}\right)$	Rainfall rate	
γ	0.0026	$\frac{\text{animal}}{\mathrm{kg}\;\mathrm{dry}\;\text{mass}}$	Plants to animal	Verwijmeren et al. (2021)
			Conversion factor	
ξ	275	$\frac{\mathrm{kg}\;\mathrm{dry}\;\text{mass}}{\text{animal year}}$	Maximal consumption rate per herbivore	Lv et al. (2019)
β	0.06	$\frac{\mathrm{kg}\;\mathrm{dry}\;\text{mass}}{m^{-2}}$	Half‐persistence parameter	Siero et al. (2019)
$\alpha_{c}$	0		Constant destocking	
$\alpha_{a}$	1		Adaptive destocking	
$\delta_{h}$	0.646	year^−1^	Such that $H_{\mathrm{eq}}=0.9H_{\max}$	
$\delta_{l}$	0.667	year^−1^	Such that $H_{\mathrm{eq}}=0.6H_{\max}$	

All parameters are fixed, taken or derived from the literature, as given in Table A1, with the exception of α and δ, our control parameters. We are considering smallstock, that is, sheep or goat (“shoat”) animal units, such that the whole flock is female (negligible number of males) and each female lambs one offspring per year on average when vegetation is at carrying capacity (V=K). Hence, the maximal per capita growth rate is 2 year^−1^, and we can solve for γ.

The mean annual precipitation rate R is chosen to be relatively low but still above the threshold for equilibrium dynamics in our coupled system of ODEs (Boone & Wang, 2007; Briske et al., 2003).
